# Genome-Wide Association Analysis for Growth-Related Traits in Indoor- and Outdoor-Cultured Juvenile Grass Carp (*Ctenopharyngodon idella*)

**DOI:** 10.3390/ani16172695

**Published:** 2026-08-30

**Authors:** Qinghua Jiang, Kefan Guo, Weijian Feng, Xiao Liang, Lina Jiang, Yao Li, Limin Yan, Jiahao Zhang, Rui Wu, Jinyu Tang, Rongxing Xia, Guoxin Wu, Fuyong Huang

**Affiliations:** 1 Institute of Hydrobiology, Zhejiang Academy of Agricultural Sciences, Hangzhou 310021, China; jqh881130@163.com (Q.J.);; 2 Zhejiang Jiaxing Yangtze River Four Major Fish Species Farm Co., Ltd., Jiaxing 314000, China; 3 College of Fisheries and Life Science, Dalian Ocean University, Dalian 116000, China; 4 College of Life Sciences, Huzhou Normal University, Huzhou 313000, China; 5 Xinchao Fisheries Development Co., Ltd. of Xinchang County, Shaoxing 312000, China; 6 Xin Hui Specialized Aquatic Species Breeding Farm, Jiaxing 314000, China

**Keywords:** *Ctenopharyngodon idella*, growth, dietary environments, GWAS, SNP, candidate gene

## Abstract

Grass carp is a key herbivorous freshwater fish, yet long-term inbreeding reduces growth rates. Current genome-wide association studies (GWAS) primarily focus on a limited range of growth traits, while intestinal morphology is likely to emerge as a promising new proxy for both growth performance and health status. We reared juvenile grass carp from one batch in indoor vs. natural aquatic vegetation groups for 100 days, measuring body weight (BW), length (BL), height (BH), and intestinal length (IL) in 200 individuals (100/group). Phenotypic differences were significant for all traits, with IL most variable. GWAS identified 95 significant SNPs, among which 21 were related to multiple traits. Candidate gene and KEGG pathway analyses linked these SNPs to myogenesis, skeletal development, immunity, calcium signaling, and stress resistance. Twelve SNPs were found to be associated with IL, including 8 multi-trait-related and 4 trait-specific ones. This suggests that IL can serve as a supplementary growth trait. There was no overlap with prior GWAS, but recurrent signals on chromosomes 1, 2, 14 (BW) and 2, 3, 14, 24 (BH) merit further study. Our findings enrich molecular resources for grass carp and highlight IL’s potential for marker-assisted breeding.

## Simple Summary

Genome-wide association studies (GWAS) are widely used to identify molecular genetic markers linking genomic variants to phenotypic traits, and are also applied in precision molecular breeding of various animals and plants. The accuracy of GWAS-derived markers improves with a richer set of reference phenotypic traits. Grass carp, a globally important commercial herbivorous freshwater fish, suffers from germplasm degradation due to prolonged inbreeding. While GWAS can help enhance its germplasm resource database, the limited diversity of available phenotypic traits may hinder this process. Changes in intestinal morphology critically affect growth and health in herbivorous fish, and may serve as a promising new indicator for assessing both growth performance and health status.

## 1. Introduction

Grass carp (*Ctenopharyngodon idella*) is a herbivorous freshwater fish species characterized by rapid growth and a relatively long period of sexual maturation [1]. Meanwhile, *C. idella* is currently the freshwater fish species with the highest aquaculture production worldwide [2,3]. However, during the long process of breeding, there is bound to be long-term inbreeding, which may lead to the degradation of germplasm and a decline in the growth rate [4]. To this end, several studies used whole-genome resequencing and genome-wide association analysis (GWAS) to conduct an in-depth analysis of genetic information related to advantageous economic traits (including growth traits, disease resistance traits, and stress tolerance traits) in grass carp, and combined marker-assisted breeding to genetically improve the economic traits of grass carp [1,2,3]. Particularly, growth traits are given special attention, such as body weight, body length, and body height, which are directly related to the economic benefits of the aquaculture industry. On the other hand, expanding and diversifying the sources of parent populations (including related populations from different geographic regions and distant hybrid populations), followed by artificial insemination, is also currently the main method for obtaining grass carp with desirable traits in aquaculture [1,4,5]. Overall, the current improvement of grass carp varieties generally focuses on the genetic expression of advantageous traits. Through modern molecular breeding techniques, grass carp varieties with advantageous traits are analyzed and screened from a genetic perspective for subsequent breeding and cultivation. However, the contribution of digestive system nutrient metabolism to fish growth appears to have been overlooked in genetic studies targeting desirable traits.

The digestive system is essential for maintaining overall health in organisms. It consists of a series of organs and structures, whose function is to process food and absorb nutrients through the processes of food intake, digestion, absorption, and excretion [6]. Each animal possesses a digestive system adapted to its specific dietary needs and environmental conditions, and most fish have a shorter and simpler digestive tract compared to mammals, including cetaceans [7]. The fish digestive system comprises the digestive tract and glands, with the intestine handling the main chemical digestion and nutrient absorption [8]. Feed quality and gut microbiota also affect intestinal absorption and digestive capacity, thereby influencing the growth and development of fish [9,10]. Additionally, the intestinal microbiota is influenced by the dietary characteristics (carnivorous, herbivorous and omnivorous) of fish and the structural characteristics of the intestine (such as intestinal length and surface area) [7,8]. Meanwhile, due to differences in dietary habits, fish exhibit significant variations in intestinal length. For instance, herbivorous fish tend to have relatively longer intestines, whereas carnivorous fish have relatively shorter ones. In addition, researchers use the relative intestinal length (RIL) to assess fish feeding habits, where RIL is defined as the total intestinal length divided by the total length (or body length); generally, the lowest RIL values are found in detritivores, intermediate values in omnivores, and the highest values in herbivores [8]. This also seems to imply that the differences in intestinal length, combined with the variations in food sources, directly affect the digestion, metabolism, and growth of fish. As a herbivorous fish, grass carp exhibits altered dietary characteristics under aquaculture conditions, where the daily feeding regimen consists primarily of artificial feed, supplemented by aquatic plants. Therefore, in the current grass carp aquaculture industry, IL and RIL may also be considered potentially complementary phenotypes for evaluating growth performance.

In this study, we first obtained a batch of grass carp larvae hatched on the same day through artificial breeding. These larvae were then divided into an indoor group (breeding in laboratory culture tanks) and a natural group (breeding in outdoor earthen ponds), and were fed twice daily. After approximately 100 days of culture (counted from the day of hatching), fish were randomly sampled from each group for phenotypic measurements (body weight (BW), body length (BL), body height (BH), and intestinal length (IL)) and sample collection (muscle tissue from the dorsal fin region). Subsequently, whole-genome re-sequencing was performed to identify the single nucleotide polymorphisms (SNPs) among individuals, and genome-wide association studies (GWAS) were conducted to screen for and investigate SNPs and potential functional genes associated with the four traits in grass carp. This study aims to further enrich the molecular genetic database of grass carp, and provide new insights and perspectives for modern molecular breeding efforts in this species.

## 2. Materials and Methods

### 2.1. Animals and Sample Collection

The parental grass carp (one male: about 7 kg (BW); one female: about 8 kg (BW)) used in this study were sourced from Zhejiang Jiaxing Yangtze River Four Major Fish Species Farm Co., Ltd. in Jiaxing, Zhejiang Province. Eggs and sperm were collected separately, followed by in vitro fertilization, and then cultured in breeding ponds (water temperature range: 26.5–27 °C; dissolved oxygen range: 5–7 mg/L; pH range: 7–8), and we obtained juvenile grass carp with a body length of approximately 0.8 cm; this work was completed on 23 May 2025. Subsequently, a portion of the larvae with good vitality (body length range: 1–2 cm) was selected for next rearing. The indoor group was reared in cylindrical culture tanks (one 1000 L tank; during the culture cycle (from June to September): water temperature range: 23–28 °C; dissolved oxygen range: 5–7 mg/L; pH range: 7–8) and fed twice daily, while the natural group was bred in outdoor earthen ponds (one pond, about 360 m^2^; during the culture cycle (from June to September): water temperature range: 24–33 °C; dissolved oxygen range: 3–8 mg/L; pH range: 7.5–8.5) and fed twice daily. Two types of feed were used: (1) a 0.3 mm diameter feed (commercial name: Tongwei Kaikoule; 38% protein content) for early feeding larvae of 1–2 cm in length; (2) a 2–3 mm diameter feed (commercial name: Tongwei 182 floating feed; 29% protein content) for feeding juveniles of 5–8 cm in length. These feeds were purchased from Tongwei Co., Ltd. (Zibo, China). During the breeding period, the total daily feeding amount was controlled at 3–5% of the total fish body weight. After approximately 100 days of culture (from 1 June 2025 to 7 September 2025), these fish (a total of 200) were randomly sampled from both groups. The fish were starved for 24 h prior to sample collection. Their phenotypic data (BW, BL, BH, and IL) were measured, and muscle tissue from the dorsal fin region was collected and stored at −80 °C, with sample weights ranging from 0.5 to 1.5 g. The entire intestine (including the foregut, midgut, and hindgut) was removed using a standardized dissection procedure. After clearing the contents, the intestine was laid flat and straightened in a dissecting tray, and its length was measured with a digital vernier caliper (deli, Ningbo, China). The above work was completed on 12 September 2025, in Hangzhou, China.

### 2.2. Whole Genome Re-Sequencing

A total of 200 fish muscle samples were used for DNA extraction and library preparation. First, DNA was extracted from the samples using Tiangen DNA Extraction Kits (Tiangen, Beijing, China). Then, the concentration and quality of each DNA sample were assessed with a NanoDrop 2000 spectrophotometer (Thermo Fisher Scientific, Wilmington, DE, USA), and DNA integrity was examined by agarose gel electrophoresis. Qualified samples were used for library construction, which involved the following steps: (1) PCR amplification of DNA samples; (2) gel electrophoresis detection and gel recovery of PCR products; (3) quality testing of recovered DNA and library construction; (4) sequencing. High-throughput sequencing was performed on the DNBSEQ-T7 platform (MGI, Shenzhen, China). The above work was completed in December 2025 at Wuhan Beina Technology Co., Ltd. (Wuhan, China).

### 2.3. SNP Discovery and Genotyping

Raw data were subjected to quality control and filtration using fastp (v1.0.1) (https://github.com/OpenGene/fastp) (accessed on 16 October 2025), and clean reads were obtained. The clean reads were mapped to the reference genome using BWA-mem2 (v2.2.1) (https://github.com/bwa-mem2/bwa-mem2) (accessed on 20 October 2025); the reference genome used was the telomere-to-telomere gapless genome of grass carp [11]. Meanwhile, GATK (v4.6.0.0) (https://github.com/broadinstitute/gatk) (accessed on 23 October 2025) was used for variant detection with hard-filtering criteria to improve prediction accuracy. Raw SNPs were then subjected to filtering using the following criteria: (1) only biallelic SNPs were retained (using VCFtools (v0.1.17) with --min-alleles 2 and --max-alleles 2); (2) variants with QUAL < 60 or minor allele frequency (MAF) < 0.05 were removed; (3) variants with genotype missing rate > 10% were excluded (--max-missing 0.8); (4) at the genotype level, those with read depth (DP) < 10 or genotype quality (GQ) < 20 were set to missing; (5) samples with overall genotype missing rate >10% were excluded; (6) samples with heterozygosity rates deviating beyond ±3 standard deviations from the mean were removed as outliers; and (7) cryptic relatedness was assessed using PLINK (v1.90) IBD estimation (PI_HAT > 0.185) to detect unexpected sample duplicates or sample swaps. No individuals were removed at this step, as all elevated PI_HAT values were consistent with the known full-sibling pedigree structure. Familial relatedness was instead accounted for via the kinship matrix incorporated into the mixed linear model. All 200 individuals passed quality control and were retained for the final GWAS analysis. Following quality control, genome-wide association analyses were conducted using a mixed linear model (MLM) to correct for population structure and familial relatedness. To address multiple testing, the Benjamini–Hochberg False Discovery Rate (FDR) procedure was applied, and SNPs with an FDR-adjusted Q-value < 0.05 were considered statistically significant. Based on the significant loci identified, the upstream and downstream 50 kb intervals were used for LD block analysis. Ultimately, high-quality SNPs were retained for subsequent analyses.

### 2.4. Population Structure Analysis

Prior to GWAS, population structure analysis was performed. ADMIXTURE (v1.3.0) (https://dalexander.github.io/admixture/index.html) (accessed on 24 October 2025) was used to infer ancestry composition from the multi-locus SNP genotype data, in order to evaluate ancestral components and subpopulation clusters. Then, GCTA (v1.93.2) (https://cnsgenomics.com/software/gcta/#Overview) (accessed on 27 October 2025) was used to perform principal component analysis (PCA), and Phylip (v3.696) (https://evolution.genetics.washington.edu/phylip.html) (accessed on 28 October 2025) was used to construct a phylogenetic tree.

### 2.5. Genome-Wide Association Study

In this study, we used EMMAX (v20120210; Efficient Mixed-Model), GEMMA (v0.98.5; two models: gemma-lm and gemma-lmm), and rMVP (v1.0.4; three models: GLM, MLM, and FarmCPU) to conduct GWAS on four growth traits (BW, BL, BH, and IL). For all models, the first ten principal components from PCA were included as fixed covariates to control for population stratification [12,13]. In the EMMAX analysis, to account for the genetic relatedness among full-sibling individuals derived from a single male × female cross, a marker-based kinship matrix was constructed using the emmax-kin utility. Specifically, the Balding–Nichols (BN) kinship matrix was estimated from the genome-wide SNP data after quality control, as the BN method provides a more accurate measure of relatedness than the identity-by-state (IBS) approach in EMMAX. This kinship matrix was then incorporated into the mixed linear model as a random-effect covariance matrix via the -k option, effectively correcting for the genetic covariance due to family structure. For GEMMA and rMVP, the default built-in kinship estimation and variance components were applied. Visualisation of the association results, including Quantile-Quantile (Q-Q) and Manhattan plots, was performed using the R package CMplot (v4.5.1) (http://CRAN.R-project.org/package=CMplot) (accessed on 29 October 2025).

### 2.6. Candidate Gene Acquisition and Functional Annotation

The reference genome of grass carp used in this study was the recently published telomere-to-telomere (T2T) gapless genome (NCBI Assembly: GCA_049919795.1; BioProject: PRJNA1251791; NGDC accession: PRJCA036158) [11]. Subsequently, we used ANNOVAR (v20200607) (https://annovar.openbioinformatics.org/en/latest/) (accessed on 30 October 2025) to search for and identify candidate genes within the 100 kb regions flanking (upstream and downstream of) the significant SNPs. This work was completed in December 2025 at Wuhan Beina Technology Co., Ltd. (Wuhan, China). Furthermore, we performed a comparative analysis (using the Excel tools in WPS Office) between the identified candidate genes and growth-related candidate genes previously reported in grass carp by other researchers.

## 3. Results

### 3.1. Statistics of Four Growth Traits

**Figure 1:**
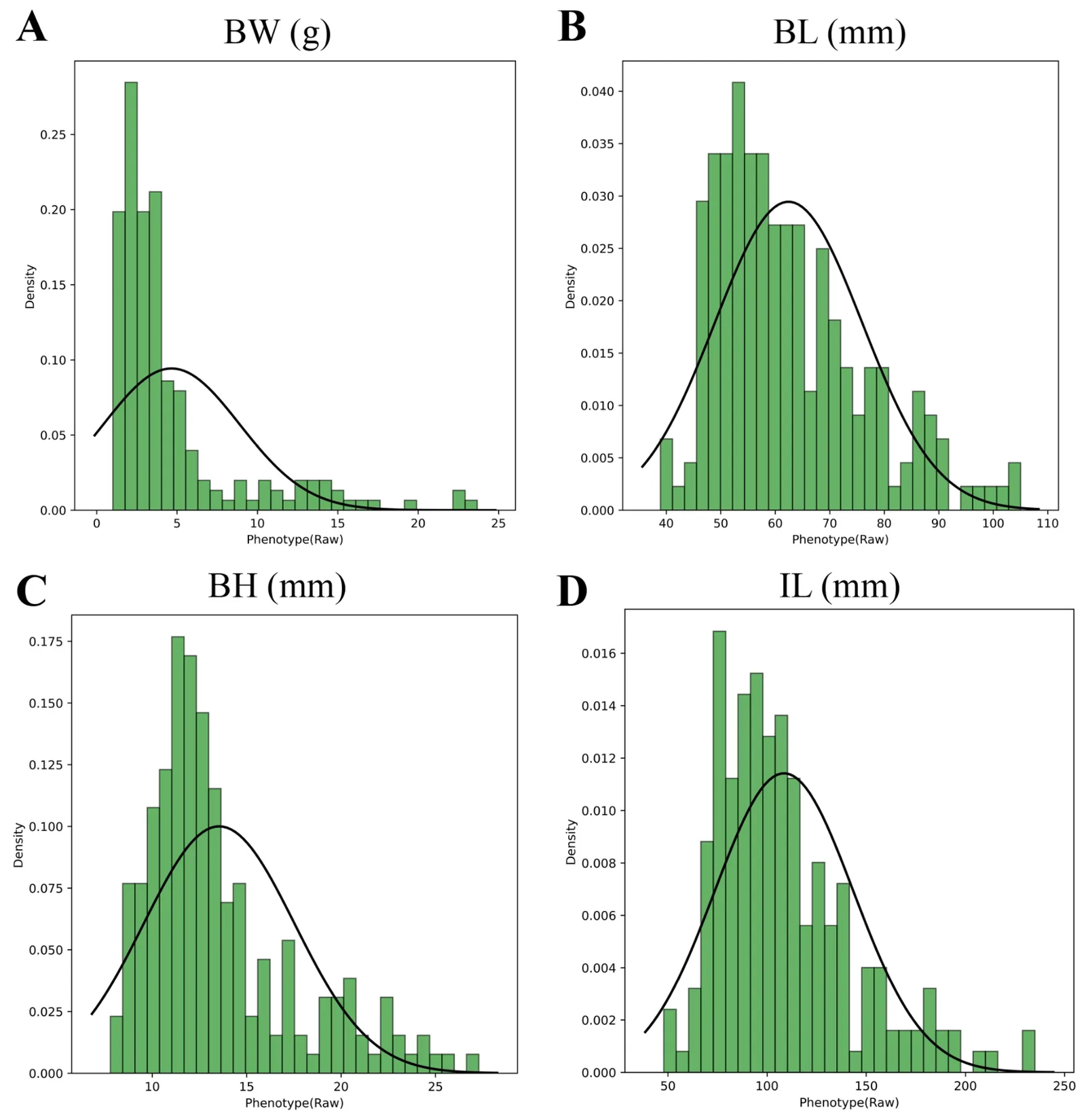
Frequency histogram of growth traits data for all the collected grass carp samples. (**A**) body weight (BW) (unit: g); (**B**) body length (BL) (unit: mm); (**C**) body height (BH) (unit: mm); (**D**) intestinal length (IL) (unit: mm). The abscissa represents the phenotypic data, while the ordinate represents the frequency of numerical distribution.

To ensure balanced sampling, we conducted random sampling for the indoor group and the natural group. The statistical results of phenotypic parameters are presented in Table 1. A total of 200 grass carp were collected from the two groups (100 fish of each group). In the indoor group, the average values of BW, BL, BH, and IL were 3.11 ± 1.59 g, 5.78 ± 0.86 cm, 1.2 ± 0.25 cm, and 9.64 ± 2.46 cm, respectively. The RIL (IL/BL) of the indoor group was 1.66 ± 0.29. In the natural group, the average values of BW, BL, BH, and IL were 6.24 ± 5.37 g, 6.71 ± 1.59 cm, 1.5 ± 0.46 cm, and 12.07 ± 3.95 cm, respectively, and the RIL was 1.78 ± 0.29. The four phenotypic datasets were analyzed using one-way analysis of variance (ANOVA) (Welch’s ANOVA was performed on groups with unequal variances) with SPSS (v24.0) (IBM, Armonk, NY, USA), and the results showed significant differences between the two groups. The distribution plots of the four phenotypic traits for all collected grass carp are shown in Figure 1. Prior to GWAS, all phenotypic data were assessed for normality by the sequencing service provider (Wuhan Beina Technology Co., Ltd.) using the Shapiro–Wilk test. BW and BL deviated significantly from normality (*p* < 0.05), while BH and IL did not (*p* > 0.05). To maintain a consistent analytical framework, rank-based inverse normal transformation (INT) was applied to all four traits, and the transformed phenotypes were used as input for all subsequent GWAS analyses. The Pearson correlation coefficients among the four phenotypes (BW, BL, BH, and IL) ranged from 0.855 to 0.939 (Table 2), and there were significant positive correlations between each pair of traits.

### 3.2. Sequencing Data, SNP Discovery, and Population Structure

**Figure 2:**
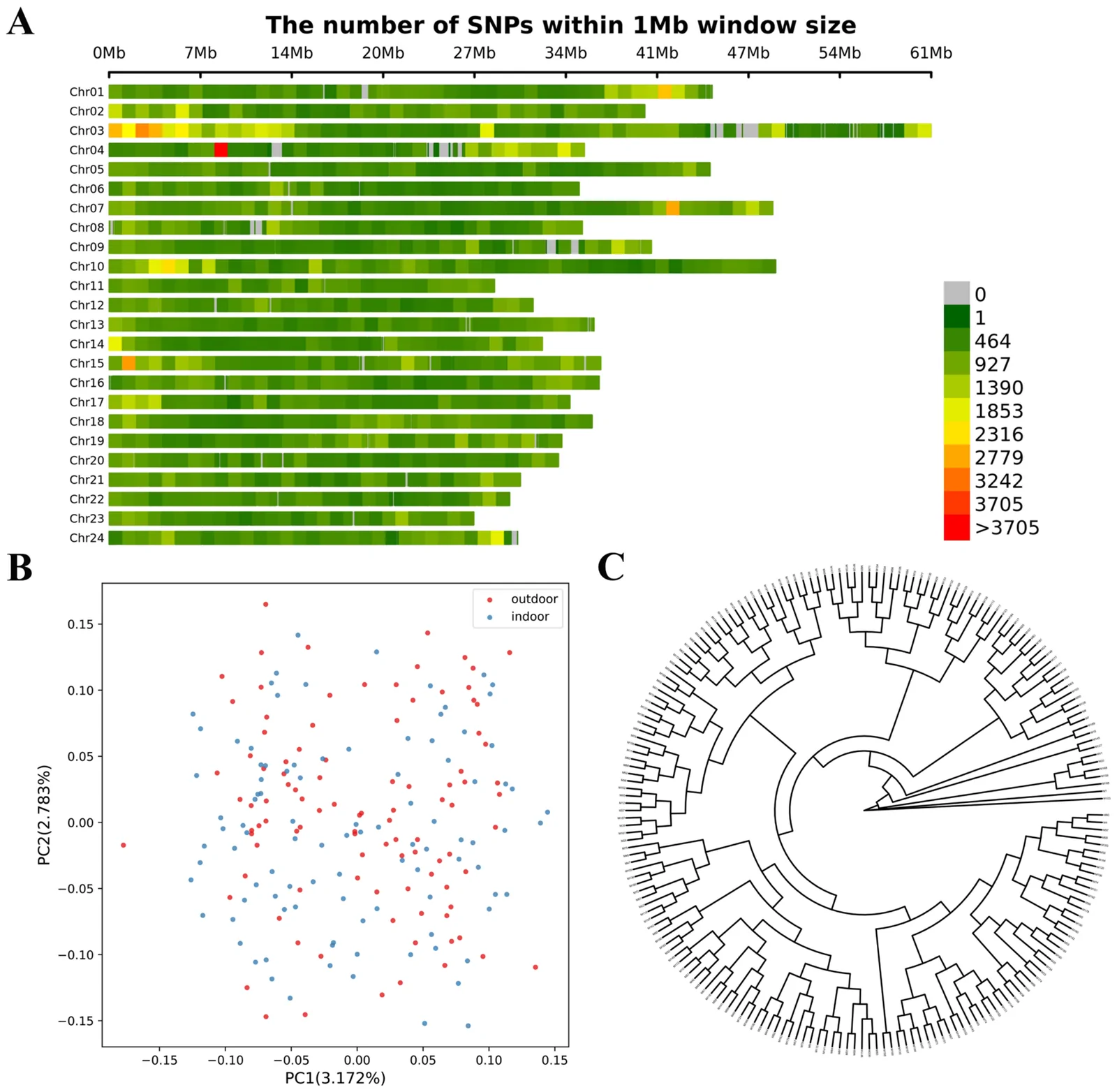
Results of SNP count statistics and population structure analysis. (**A**) The chromosomal distribution of high quality SNPs within 1 Mb windows in grass carp. (**B**) Principal component analysis of the experimental population. PC1 and PC2 represent the first two principal components that capture the largest and second-largest directions of genetic variation among all samples, respectively. The corresponding explained variation percentages reflect the degree of genetic differentiation across the entire population. (**C**) the phylogenetic tree result of the experimental population.

In this study, a total of 2097.81 Gb of raw data were obtained, and after detection and filtering, 2080.3 Gb of clean data were obtained. The filtered sequencing data showed an average Q20 of 99.54%, an average Q30 of 98.15%, and an average GC content of 38.32%. For the 200 samples, alignment rates ranged from 99.62% to 99.86%, and reference genome coverage ranged from 97.58% to 98.42% (the reference genome was the grass carp telomere-to-telomere (T2T) genome released in March 2025) [11]. The average sequencing depth ranged from 9.91× to 16.43×. After filtering, a total of 6,909,223 SNPs were retained for subsequent analysis. The optimal K value was determined according to the ADMIXTURE output (Figure S1). Figure 2A shows the density distribution of high-quality differential SNPs on chromosomes within 1 Mb windows. Principal component analysis (PCA) was performed using the genome-wide SNP data to assess population structure. The first two principal components (PCs) explained 3.172% and 2.783% of the total variance, respectively (cumulative 5.955%), indicating low genetic differentiation between the indoor group and the natural group (Figure 2B). Given this relatively low explanatory power, we included the first ten PCs as covariates in all GWAS models to more comprehensively control for potential population stratification and family structure. This approach follows the standard pipeline recommended by our sequencing service provider (Wuhan Beina Technology Co., Ltd.) and is consistent with common practice in GWAS. The phylogenetic tree (Figure 2C) further indicated that the 200 fish remained clustered together.

### 3.3. GWAS on the Growth Traits

**Figure 3:**
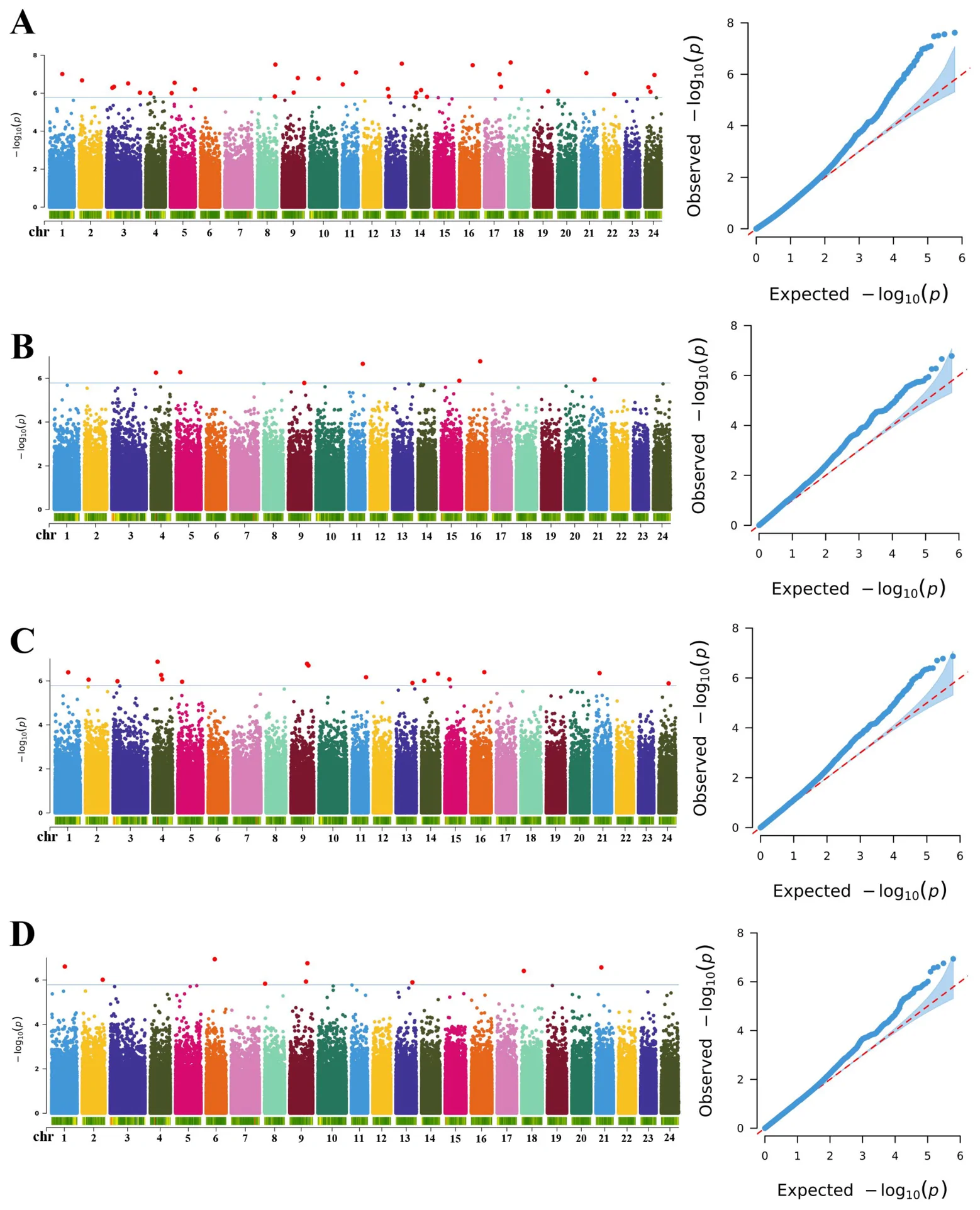
GWAS for growth-related traits in grass carp. (**A**) Manhattan and Q-Q plots for BW; (**B**) Manhattan and Q-Q plots for BL; (**C**) Manhattan and Q-Q plots for BH; (**D**) Manhattan and Q-Q plots for IL. The dotted line represents the significance threshold (5.8).

In this study, we applied multiple GWAS models (EMMAX, rMVP, and GEMMA) to identify loci associated with growth traits. For each model, we adopted a suggestive genome-wide significance threshold of P = 1/N, where N is the number of SNPs retained after quality control. Specifically, N = 611,115 for EMMAX and rMVP, giving P = 1.64 × 10^−6^ (−log_10_(P) = 5.8), and N = 391,421 for GEMMA, giving P = 2.55 × 10^−6^ (−log_10_(P) = 5.59). This threshold was chosen for three reasons: (i) LD pruning substantially reduced marker dependency, (ii) growth traits in grass carp are polygenic, making a stringent Bonferroni correction overly conservative, and (iii) this threshold is widely used in aquaculture GWAS for initial candidate discovery. To further control false positives, we applied Benjamini–Hochberg FDR correction (q < 0.05). Only SNPs that exceeded both the suggestive threshold and the FDR cutoff, and were consistently detected in at least two models, were considered candidate significant loci. We evaluated multiple GWAS models, including EMMAX, GEMMA, and rMVP. The results from these models were not entirely concordant, which is expected given their different underlying algorithms. For presenting the final results, we selected EMMAX because it is computationally efficient for large-scale datasets, effectively corrects for population structure, and has been widely used in previous grass carp GWAS studies. We do not claim that EMMAX is objectively superior to the other models; rather, it was chosen for its practical advantages. Using this approach, we identified 95 candidate significant SNPs that met the above criteria (Table S1). These loci are considered priority candidates for growth-related traits and merit further functional validation. The corresponding Manhattan and Q-Q plots are shown in Figure 3. The genomic inflation factors (λ) were 1.298, 1.175, 1.195, and 1.144 for BW, BL, BH, and IL, respectively, indicating acceptable model fit.

A total of 85 SNPs were significantly associated with BW, covering 23 chromosomes (Figure 3A). After excluding 20 multi-trait-related SNPs, 65 specific SNPs remained, corresponding to 79 candidate genes. The KEGG pathway annotation-based comparison revealed enrichment in insulin/MAPK signaling, calcium signaling, the Hippo pathway, and autophagy (mTOR), with featured genes including INSR, OCK1, and CACNA1C, all of which were related to growth, fat deposition, and muscle development. Ten SNPs showed significant association with BL, spanning 9 chromosomes (Figure 3B). Only one specific SNP (Chr01_32275869) was identified, with the predicted functional gene CFAP58, which may function in cilium/flagellum structure and activity (ko03037). A total of 22 SNPs were significantly associated with BH, covering 14 chromosomes (Figure 3C). Among them, five specific SNPs were identified, with predicted genes including VPREB, CD179a, CD84, CD21, and KIF4. The involved KEGG pathways included cell adhesion molecules (map04514), membrane trafficking (map04131), cytoskeleton in muscle cells (map04820), and Toll and Imd signaling (map04624), most of which are implicated in skeletal growth and body shape development. Another 12 SNPs were significantly associated with IL, covering 10 chromosomes (Figure 3D), with four specific SNPs identified. Predicted genes included EEF1D, CD276, MECP2, and OXR1, potentially involved in peptide chain elongation, cell adhesion molecules (CAMs), lysosome function, and the p53 signaling pathway.

Additionally, 21 multi-trait-related SNPs across 14 chromosomes corresponded to 29 candidate genes (such as WBP1, FLNC, SYT4), with enriched KEGG pathways including focal adhesion, N-glycan biosynthesis, neurotrophin signaling, and cGMP-PKG signaling—pathways involved in cytoskeletal remodeling, energy metabolism, neurodevelopment, and immune regulation. Overall, specific SNPs showed more confined functional enrichment, whereas multi-trait-related SNPs covered a broader range of pathways.

### 3.4. Candidate Gene Prediction and Functional Annotation

**Figure 4:**
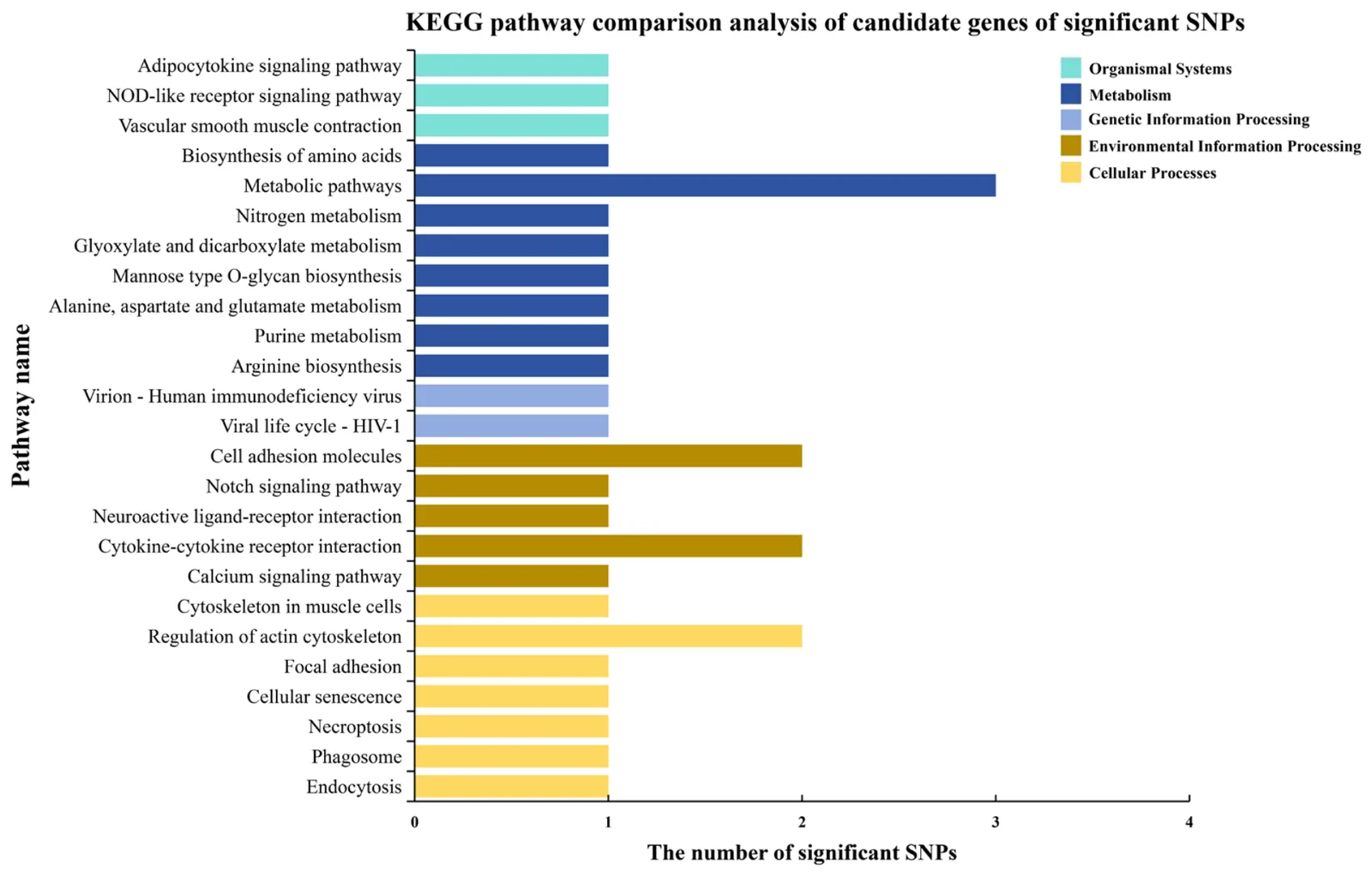
KEGG pathway comparison analysis of candidate genes for the significant SNPs associated with multiple traits. The abscissa denotes the count of significant SNPs, the ordinate denotes the pathway names, and the different bar colors indicate the first-level KEGG pathway categories.

Through comparative analysis with the chromosomal sequence positions of the reference genome, genes containing or closest to the significant SNPs (within 100 kb up- and downstream of the SNPs) were regarded as candidate genes associated with growth traits. In this study, a total of 118 candidate genes were identified around 95 SNPs associated with the four growth traits (Table S1). Further analysis revealed that 21 SNPs were simultaneously associated with multiple traits (Table 3). Among them, 3 SNPs (Chr04_7212791; Chr09_28444185; Chr21_9415983) were associated with four growth traits (BW-BL-BH-IL), 6 SNPs (Chr01_23255436; Chr13_28752922; Chr04_8637746; Chr05_7613637; Chr11_24865131; Chr16_24161648) were associated with three growth traits (BW-BH-IL; BW-BL-BH), and 12 SNPs (Chr02_2132254; Chr18_3555396; Chr03_974669; Chr04_15035058; Chr04_16782891; Chr14_28928243; Chr14_4698999; Chr15_7993310; Chr24_17059758; Chr10_16930075; Chr15_32531357; Chr09_30872441) were associated with two growth traits (BW-IL; BW-BH; BW-BL; BH-IL). Furthermore, we conducted the KEGG pathway comparison analysis on the candidate genes corresponding to these 21 SNPs that are associated with multiple traits (Figure 4). The results revealed that the 17 candidate genes linked to 11 SNPs were mapped to 25 KEGG pathways, such as Metabolic pathways (SNPs: Chr21_9415983, Chr16_24161648, and Chr18_3555396), Cell adhesion molecules (Chr04_8637746 and Chr03_974669), Cytokine-cytokine receptor interaction (Chr03_974669 and Chr04_15035058), Regulation of actin cytoskeleton (Chr04_8637746 and Chr04_16782891). Meanwhile, we reviewed other published GWAS studies on growth-related traits in grass carp [1,3], and compiled all significant SNPs (including the 140 SNPs reported previously and 95 SNPs newly identified in this study) and their candidate genes for BW, BL, and BH (Table S2). Regrettably, we did not identify any significant overlapping information, which may require further in-depth exploration in subsequent studies.

## 4. Discussion

In this study, phenotypic trait analysis showed significant differences between indoor and natural groups, with IL exhibiting the most pronounced effect (Table 1), supporting that dietary environment is associated with growth traits, particularly intestinal parameters. However, PCA (Figure 2B) revealed minimal group separation, likely due to the common origin of fry (Figure 2C) or insufficient sample size. Furthermore, We acknowledge that the rearing environment was not modelled as a covariate in our GWAS, and thus the potential confounding effect of environmental variation cannot be completely ruled out; independent validation across additional families and culture systems is warranted to confirm our findings. Following filtering and analysis, we identified 95 significant SNPs (Figure 3, Table S1). Of these, 21 SNPs were related to multiple traits (Table 3), distributed across traits (BW-BL-BH-IL: 65.8%, 7.8%, 17.1%, 9.3%), suggesting IL as a suitable GWAS trait. Candidate gene prediction and functional annotation yielded 118 candidate genes in total, with 29 linked to the 21 multi-trait-related SNPs (Table S1). Comparison with previous grass carp GWAS studies (Table S2) did not reveal identical mutation sites. This phenomenon may be attributable to a range of factors, such as genetic differences among the broodstock, environmental variability in culture conditions, divergent analytical approaches, and distinctions in the traits examined and the corresponding treatment methods [14,15,16]. These factors collectively may exert either direct or indirect influences on the experimental outcomes. Nevertheless, we have identified some noteworthy findings: significant single nucleotide polymorphisms (SNPs) associated with body weight (BW) have been repeatedly reported on chromosomes 1, 2, 10, 14, 17, and 22; those related to body height (BH) are predominantly concentrated on chromosomes 2, 3, 14, and 24; whereas those associated with body length (BL) have been found only on chromosome 4. This suggests that these chromosomes may be more frequently involved in growth-related traits and thus warrant further investigation.

BW-specific SNPs were the most abundant among the four traits (65 SNPs), covering 23 chromosomes. They corresponded to 79 candidate genes, including several functionally important ones such as INSR (involved in insulin, MAPK, Ras, and FoxO signaling), CACNA1C (calcium signaling, cardiac contraction, adrenergic signaling in cardiomyocytes), PDPK1 (FoxO, insulin, PPAR, and sphingolipid signaling), ROCK1 (vascular smooth muscle contraction, actin cytoskeleton regulation, TGF-β signaling, axon guidance, chemokine signaling, and focal adhesion), and COL6A (cell adhesion, ECM-receptor interaction, PI3K-Akt signaling, and protein digestion/absorption). This suggests that different rearing environments most directly affect body weight. BH-specific SNPs were second most frequent (5 SNPs), with 9 candidate genes; among them, three (CD179a, CD84, CD2) are involved in cell adhesion molecules (map04514), and one (ANK2) participates in Toll/Imd signaling (map04624) and cytoskeleton in muscle cells (map04820). This findings consistent with skeletal development influencing body height. Only one BL-specific SNP was identified (the total number of BL-specific SNPs is 10), whereas IL-specific SNPs numbered 4 (out of 12 total IL-associated SNPs), potentially representing novel discoveries. The IL-specific SNPs predicted candidate genes including EEF1D (peptide elongation/protein metabolism), B7H3 (CD276, cell adhesion molecules), MECP2 (epigenetic regulator), and OXR1 (lysosome and p53 signaling). Given that intestinal health relies on a delicate balance between cell renewal and apoptosis, B7H3, MECP2, and OXR1 may be particularly relevant among these candidates.

On the other hand, we performed a comparative analysis of KEGG pathways for the 21 multi-trait-related SNPs (Figure 4). The result revealed that 17 candidate genes linked to 11 SNPs (Chr04_7212791, Chr21_9415983, Chr13_28752922, Chr04_8637746, Chr11_24865131, Chr16_24161648, Chr18_3555396, Chr03_974669, Chr04_15035058, Chr04_16782891, and Chr15_7993310) were mapped to 25 KEGG pathways. These pathways covered five KEGG pathway categories and included some important ones, such as cytoskeleton in muscle cells, regulation of actin cytoskeleton, and the Notch signaling pathway. Among them, the candidate gene of Chr04_7212791 was FLNC, some studies have indicated that FLNC plays a crucial role in muscle development and the maintenance of muscle structural integrity [17,18,19]. The candidate gene of Chr09_28444185 was VWA8. In zebrafish, deletion or mutation of the VWA8 gene will lead to early developmental delay, motor impairment, and skeletal abnormalities [20]. The candidate gene of Chr21_9415983 was GLUL (glnA), which has been proven to play a significant role in regulating osteogenic differentiation and bone development [21]. These three SNPs were significantly associated with all growth traits examined (BW-BL-BH-IL). Notably, the KEGG functional annotations of their candidate genes appear to provide tentative support for this experimental inference. In addition, the other 18 SNPs also merit significant attention. The candidate gene of Chr04_8637746 (associated with three growth traits: BW-BL-BH) was CD163 and ITGAM (CD11b), both of which are critically involved in the inflammatory process. ITGAM (CD11b) serves as a major phagocytic receptor on myeloid cells, triggering phagocytosis after binding to ligands such as fibrinogen, and concurrently acts as a negative regulatory factor in immunosuppression [22,23]; CD163, a macrophage scavenger receptor, is essential for both acute and chronic inflammatory immune responses [24,25]. Moreover, MCU is a mitochondrial Ca^2+^ uniporter that regulates mitochondrial calcium uptake, and mutations or deletions of this gene can cause acute loss of matrix calcium ions, thereby increasing fatty acid oxidation (FAO) and alleviating the obese state in mice [26]; MICU1 is critically involved in the regulation of mitochondrial calcium homeostasis, maintenance of mitochondrial integrity, and control of redox balance, in addition, it confers enhanced cold tolerance in fish through the preservation of membrane potential, the attenuation of cold-induced ROS generation, and the suppression of both ferroptosis and apoptosis [27]. These two genes were the candidate genes of Chr13_28752922 (associated with three growth traits: BW-BH-IL), which also implied that Chr13_28752922 is likely to be associated with the body’s inflammatory response. The candidate gene at Chr04_15035058 is LEP (leptin), an important hormone associated with obesity and growth processes, which plays a critical role in regulating appetite, lipid metabolism, and overall growth in fish [28]. Although these SNPs exhibited a random distribution across the 24 chromosomes of grass carp, the candidate genes with which they were associated are non-randomly distributed and were predominantly enriched in key biological processes, including muscle development, bone development, immune inflammation, calcium signaling pathways, and stress resistance regulation. In summary, the candidate genes for multi-trait-associated SNPs show a wider and more complete functional spectrum compared with those for single-trait-specific SNPs, which seems to align with the concept of pleiotropy. However, further intensive studies are still needed on the discriminative features of these multi-trait SNPs and the functional roles of their candidate genes, so as to determine molecular markers appropriate for application in molecular selective breeding of grass carp.

## 5. Conclusions

In this study, we conducted a genome-wide association analysis in two groups of grass carp reared under different feeding conditions to identify SNPs and candidate genes associated with four growth traits. We acknowledge that the suggestive threshold used here is less stringent than a conventional Bonferroni correction; however, given the polygenic architecture of growth traits and the use of an LD-pruned SNP set, we consider this approach appropriate for prioritizing candidate loci for downstream validation while minimising false negatives. Future studies with larger sample sizes and independent populations will be required to confirm these associations. A total of 95 significant SNPs were identified as closely associated with the four growth traits. Among these, 21 SNPs were associated with multiple growth traits and implicated in many important biological processes, including myogenesis, skeletal development, inflammatory immune responses, calcium signaling, and stress resistance. Notably, 12 SNPs showed associations with intestinal length (IL), including 8 multi-trait-related SNPs and 4 IL-specific SNPs. This observation provides initial evidence that IL may serve as a potentially complementary phenotype for growth performance, yet the features of the identified SNPs merit further exploration. In summary, our results further enrich the theoretical foundation and genetic background for molecular marker-assisted breeding research on grass carp.

## Figures and Tables

**Table 1:** Summary of statistical analyses for growth traits in all grass carp samples. Footnote: Body weight (BW), unit: g; body length (BL), unit: cm; body height (BH), unit: cm; intestinal length (IL), unit: cm; relative intestinal length (RIL).

Group (Sample Number)	The Indoor Group (*n* = 100)			The Natural Group (*n* = 100)		
Phenotype Statistics	Average Value	Max Value	Min Value	Average Value	Max Value	Min Value
BW (g)	* 3.11 ± 1.59	9.64	1.14	6.24 ± 5.37	23.67	1.01
BL (mm)	* 5.776 ± 0.856	7.951	3.893	6.711 ± 1.591	10.502	3.981
BH (mm)	* 1.203 ± 0.247	1.981	0.778	1.504 ± 0.463	2.729	0.807
IL (mm)	** 9.638 ± 2.457	19.401	4.857	12.069 ± 3.954	23.502	4.793
RIL (IL/BL)	* 1.66 ± 0.29	2.47	1.02	1.78 ± 0.29	2.54	0.99

Footnote: “*” represents a significant difference between the two groups, and *p* < 0.05, “**” represents *p* < 0.01.

**Table 2:** The Pearson correlation coefficients among four phenotypes for experimental grass carp samples.

Phenotype	BL	BH	BW	IL
BL	1	0.917 **	0.895 **	0.855 **
BH	0.917 **	1	0.939 **	0.900 **
BW	0.895 **	0.939 **	1	0.873 **
IL	0.855 **	0.900 **	0.873 **	1

Footnote: “**” represents *p* < 0.01, and also means significant positive pairwise correlations.

**Table 3:** Concise summary table for significant SNPs linked to multiple growth traits.

SNP Position	Chr	Candidate Gene Name	Simultaneously Associated Growth Traits
Chr04_7212791	4	FLNC	BW; BL; BH; IL
Chr09_28444185	9	VWA8	BW; BL; BH; IL
Chr21_9415983	21	GLUL/glnA	BW; BL; BH; IL
Chr01_23255436	1	WBP1	BW; BH; IL
Chr13_28752922	13	MCU, MICU1	BW; BH; IL
Chr04_8637746	4	ANKRD50, CD163, ITGAM/CD11b	BW; BL; BH
Chr05_7613637	5	MCAM/CD146	BW; BL; BH
Chr11_24865131	11	ACAP	BW; BL; BH
Chr16_24161648	16	SEMA6, E4.6.1.2, TNFAIP8	BW; BL; BH
Chr02_2132254	2	—	BW; IL
Chr18_3555396	18	B3GAT1	BW; IL
Chr03_974669	3	CD4	BW; BH
Chr04_15035058	4	LEP, SND1	BW; BH
Chr04_16782891	4	PPP1R12A/MYPT1	BW; BH
Chr14_28928243	14	PCDHB, FAT4, PCDHD2, PCDHA	BW; BH
Chr14_4698999	14	PCDHD1	BW; BH
Chr15_7993310	15	NUMBL	BW; BH
Chr24_17059758	24	SYT4	BW; BH
Chr10_16930075	10	CD84	BW; BL
Chr15_32531357	15	AGMO	BW; BL
Chr09_30872441	9	KCNJ3/KIR3.1	BH; IL

Footnote: “—” indicates that no candidate genes meeting the search criteria were identified.

## Data Availability

The raw data and other data that support the findings of this study are available from the corresponding author upon reasonable request.

## Supplementary Materials

The following supporting information can be downloaded at https://www.mdpi.com/article/10.3390/ani16172695/s1: Table S1. Summary table of all significant SNPs identified in this study that are associated with the four growth traits. Footnote: The number of asterisks (*) indicates the number of growth traits with which the SNP is significantly associated. Table S2. Summary table of significant SNPs identified in recent studies that are associated with three growth traits (BW, BL, and BH). Figure S1. The analysis results by using ADMIXTURE. (A) the result of CV-error; (B) comparative analysis of individual ancestral components under varying K values. Each bar represents an individual sample. Colors denote different ancestral populations, and the proportion of each color indicates the estimated ancestry proportion contributed by that population to the sample.
