# Supplementary material for: Genome-Wide Association Analysis for Growth-Related Traits in Indoor- and Outdoor-Cultured Juvenile Grass Carp (Ctenopharyngodon idella)
Source: Animals (Basel). 2026 Aug 30;16(17):2695. doi: 10.3390/ani16172695 (PMC13564318; doi:10.3390/ani16172695)
Supplement: Supplementary file 1 [file animals-16-02695-s001.zip › Supplementary material legend.pdf]

Supplementary material information:

Table S1. Summary table of all significant SNPs identified in this study that are associated with the four growth traits. Footnote: The number of asterisks (*) indicates the number of growth traits with which the SNP is significantly associated.

Table S2. Summary table of significant SNPs identified in recent studies that are associated with three growth traits (BW, BL, and BH). Footnote: The number of asterisks (*) indicates the number of growth traits with which the SNP is significantly associated.

Figure S1. The analysis results by using ADMIXTURE. (A) the result of CV-error; (B) comparative analysis of individual ancestral components under varying K values. Each bar represents an individual sample. Colors denote different ancestral populations, and the proportion of each color indicates the estimated ancestry proportion contributed by that population to the sample.
